# Correction: Does China Pakistan Economic Corridor become an avenue to achieve sustainable development goal no. 2 (food security) in Pakistan: Under the condition of COVID-19?

**DOI:** 10.1371/journal.pone.0282686

**Published:** 2023-03-01

**Authors:** The PLOS ONE Editors

## Notice of republication

This article was republished on February 21, 2023, to address a file issue. The article’s Data Availability statement has also been updated. Please download this article again to view the correct version.
